# Skin to calyx distance is not a predictive factor for miniaturized percutaneous nephrolithotomy outcomes

**DOI:** 10.1590/S1677-5538.IBJU.2016.0291

**Published:** 2017

**Authors:** Faruk Ozgor, Onur Kucuktopcu, Burak Ucpinar, Fatih Yanaral, Murat Binbay

**Affiliations:** 1 Department of Urology, Haseki Training and Research Hospital, Istanbul, Turkey

**Keywords:** Obesity, Nephrostomy, Percutaneous, Kidney Calculi

## Abstract

**Objective:**

To evaluate the predictive value of the distance from skin to calyx (SCD) on the outcome and complication rates of patients undergoing mPNL.

**Materials and Methods:**

Patient’s charts, who had undergone mPNL between June 2012 and June 2015, were analyzed retrospectively. Patients who had a preoperative computerized tomography (CT) were enrolled into the study. Two separateurologists evaluated the CT scans and calculated the SCD defined as the distance between the skin and surface/lateral edge of the calyx, which was the preferred site of entry for percutaneous access. The average value of the two measurements was included inthe final analysis to avoid bias. The mean SCD was 75mm. According to the median SCD value, patients were divided into two groups: group 1 (SCD ≤75) and group 2 (SCD >75).

**Results:**

A total of 140 patients and 130 patients were enrolled in groups 1 and 2, respectively. The mean operation time and the mean fluoroscopy time was significantly longer in group 2 (p:0.004 vs. p:0.021). The rate of blood transfusion was significantly higher in group 1 (6 patients). None of patientsin group 2required blood transfusion (p:0.017). Stone-free status after a single session of mPNL was 67.1% in group 1 and 75.4% in group 2 (p:0.112). After additional procedures, stone-free rates increased to 84.3% and 85.4% in group 1 and group 2, respectively (p:0.802).

**Conclusion:**

Our study demonstrated that longer SCD was not a predictive factor for stone-free rates after mPNL. However, SCD over 75mm was associated with longer operation time and fluoroscopy time with lower rates of transfusion.

## INTRODUCTION

Percutaneous nephrolithotomy (PNL) is a well-established surgical optionfor renal stone(s) larger than 2cm and staghorn calculi. Satisfactory stone-free rates can be achieved with PNL. Yet, the procedure itself bears some serious potential complications including bleeding,which may require blood transfusion, adjacent organ injury and septicemia (1). Conventional PNL is performed using a 24F to 30F nephrostomy tract and the use of larger size instruments have been associated with unfavorable outcomes (2). Recent advances in technology have enabled the design of instruments with smaller diameters to use in PNL. Miniaturized percutaneous nephrolithotomy (mPNL) is defined asPNL performed by using an instrument with an access sheath of 12-20F diameter(3).

Factors influencing the outcome of PNL including stone burden, renal abnormalities, surgeon experience and obesity had been clearly described (4). In obese patients, access to the *pelvi-calyceal system*and the appropriate dilatation of the tract presents a challenge for the surgeon. Moreover, in obese patients, the inadequate length of the working sheath and working instrumentshave an adverse effect on PNL outcomes. Taking into consideration the differences in body types and body fat distribution among people and races, some authors suggested that the distance from skin to calyx (SCD) is amore predictive factor than body mass index on PNL outcomes (5).

Factors affecting the mPNL outcomes are still being investigated and the role of SCD has not been previously evaluated. To our knowledge, this is the first study that investigates the effect of SCD onthe outcome and complication rates of patients undergoing mPNL.

## MATERIALS AND METHODS

After approval from the ethics committee, a retrospective*chart*review of*a consecutive series of patients*undergoing mPNL in a tertiary academic center between June 2012 and June 2015 was analyzed. Every patient included in the study had undergone a preoperative computerized tomography (CT) scan and a follow-up imaging 3 months after the mPNL operation. Patients under 18 years of age, withkidney abnormalities, with no available preoperative CT scans and with a history of PNL procedurewith multiple accesses were excluded from the study.

**Figure 1:**
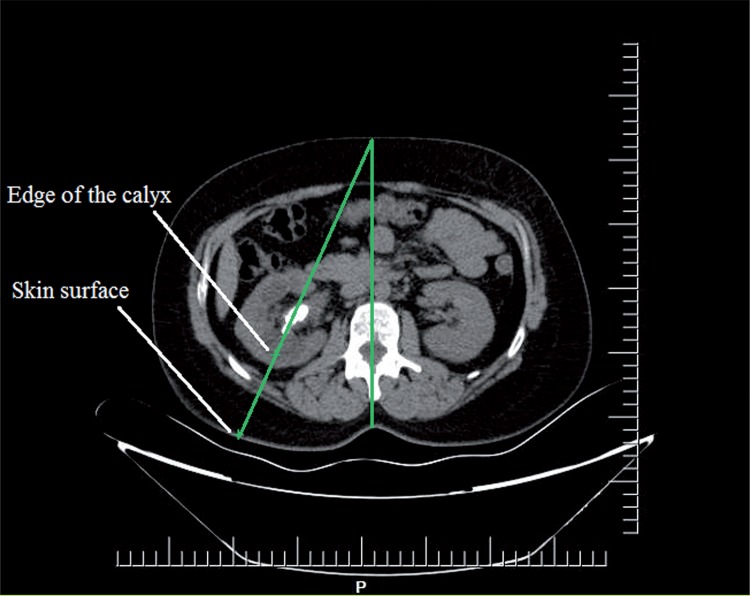
Example of an axial image from a CT scan, demonstrating the way 'Skin to calyx distance'calculated.

Kidney and stone characteristics of every patient were evaluated with non-contrast computerized tomography, preoperatively. Complex stones were defined as those located in the pelvis and in at least one calyx or multiple calyces.For each patient, stone size and SCD were measured by two separate urologists to avoid bias. The average value between the two measurements was taken for final analysis. The distance was calculated by drawing a vertical line from the spinous process to the anterior abdominal wall and a second line starting from the anterior abdominal wall and traversing the point of the calyceal edge. The SCD was defined as the distance between the skin and surface/lateral edge of the calyx, which was the preferred site of entry for percutaneous access (Figure-1). While calculating the SCD, Hounsfield unit difference between renal parenchyma and pelvicaliceal system was taken into account to identify the exact point of differentiation between calyx and parenchyma. In our study, the calculated mean SCD was 75mm. According to the median SCD value, patients were stratified into two groups: group 1 (SCD ≤75) and group 2 (SCD >75).

### Surgical technique

In the lithotomy position under general anesthesia, a 5Fr ureteral catheter was inserted. In the prone position, the calyceal system was visualized using contrast media and access was performed to appropriate calyx using an 18G needle under the C-armed scopy unit. A 0.035-inch hydrophilic guide-wire was delivered into the pelvicalyceal system. The access tract was dilated using Amplatz dilatators, then an 18- or 20Fr Amplatz sheath was inserted into the pelvicalyceal system. Stone fragmentation was performed with laser or ultrasonic lithotripter and stone extraction forceps was used for stone removal. After completion of the procedure, nephrostomy tube was placed under fluoroscopy if necessary. Operation time was calculated as the time from access to the preferred calyx to the insertion of the nephrostomy tube.

On the first postoperative day, a kidney-ureter-bladder radiogram was performed to evaluate the success.On the 3^rd^ postoperative month, stone-free status was reassessed in the outpatient setting with non-contrast abdominal CT. Patients with complete stone clearence and patients with residual fragments under 4mm were accepted as stone free.

For statistical analysis, values were evaluated as numbers, means, percentages and intervals. Numbers and percentages were compared using the Chi-square test. Before the comparison of means of values, the values were evaluated for homogenity. Homogenously distributed values were compared using Student T test and heterogenously distributed values were compared using Mann Whitney U test.

## RESULTS

According to the study design, 140 patients with SCD ≤75mm (range, 48-75mm) and 130 patients with SCD >75mm (range, 76-126mm) were enrolled into group 1 and group 2, respectively. Preoperative characteristics including gender, age, stone size and stone location were similar between the groups (p:0.823, p:0.129, p:0.143 and p:0.077, respectively). Also, the mean BMI was comparable between the groups (p:0.090). Preoperative charecteristics of the two groups are summarized in Table-1.

**Table 1:** Comparison of preoperative demographics.

	Skin to calyx distance		
	
	≤75mm	>75mm	P value
Number	140	130	
**Gender**			**0.823**
Male	88	80	
Female	52	50	
Mean age (years)	43.09±12.5	45.4±12.4	0.129
Mean body mass index (kg/m^2^)	25.7±3.5	26.6±5.3	0.090
Mean stone size (mm)	21.7±5.8	22.8±6.0	0.143
**Stone location**			**0.077**
Complex stone	60	57	
Pelvis	31	34	
Lower	40	30	
Middle	8	6	
Upper	1	3	
**Operation side**			**0.745**
Left	76	68	
Right	64	62	
**Degree of hydronephrosis**			**0.682**
0	6	4	
1	72	66	
2	54	54	
3	6	6	
4	2	0	
**Previous renal stone treatment**			**0.853**
SWL	22	21	
PNL	14	17	
Open renal stone surgery	11	13	

In both groups, the lower pole was the most preferred location for access. The mean operation time was 91.6±37.7min. in group 1 and 105.8±41.9 min. in group 2 (p:0.004). The mean fluoroscopy time was significantly longer in group 2 (p:0.021). The mean hemoglobin drop was higher in group 1 but the difference was not statistically significant (p:0.178). The mean duration of hospitalization time was comparable between the groups (p:0.404) (Table-2).

**Table 2:** Comparison of perioperative findings.

	Skin to calyx distance		
	
	≤75mm	>75mm	P value
Number	140	130	
Mean operation time (minutes)	119.6±39.1	131.8±43.1	**0.002**
Mean fluoroscopy time (minutes)	5.0±3.7	6.1±3.4	**0.021**
**Access location**			**0.051**
Lower	126	114	
Middle	14	10	
Upper	0	6	
Number of intercostal access	0	4	**0.037**
Mean hemoglobin drop (g/dL)	1.02±1.50	0.78±1.1	0.178
Mean hospitalization time (hours)	68.8±25.8	65.6±36.0	0.404

Complications as evaluated by the Clavien system were similar between the groups (p:0.155). Requirement of postoperative JJ insertion was the most common complication in both groups (9 patients in group 1 and 13 patients in group 2). Fever requiring antibiotic therapy was observed in 4 patients (2 patients in group 1 and 2 patients in group 2). When the complications were separately evaluated, the rate of bleeding requring blood transfusion was significantly higher in group 1 and occurred in 6 patients. In two of the 6 patients, angioembolisation was required. In group 2, no patients required blood transfusion (p:0.017). No pulmonary complications and Clavien grade 4 or 5 complications were encountered in both groups.

Stone-free status after a single session of mPNL was 67.1% and 75.4% in group 1 and group 2, respectively (p:0.112). Spontaneous passage of residual fragments occurred in 12 patients. Additional procedures including SWL, F-URS and mPNL were performed to 20 patients in group 1 and to 12 patients in group 2. After additional procedures, stone free-rates increased to84.3% and 85.4% in group 1 and group 2, respectively (p:0.802) (Table-3).

**Table 3:** Comparison of postoperative results and complications.

	Skin to calyx distance		
	
	≤75mm	>75mm	P value
Number	140	130	
Post operative complications according to Clavien System			0.155
**Grade 2**			
UTI	2 (1.4%)	2 (1.5%)	
Transfusion requirement	6 (4.3%)	0	
**Grade 3a**			
Postoperative JJ insertion without anesthesia	7 (5%)	10 (7.6%)	
**Grade 3b**			
Postoperative JJ insertion with anesthesia	2 (1.4%)	3 (2.3%)	
Angioembolisation requirement	2 (1.4%)	0	
Stone free status	94 (67.1%)	98 (75.4%)	
Additional procedures			0.472
Spontane passage	6	6	
SWL	8	2	
URS/f-URS	4	4	
mPNL	8	6	
Stone free status after additonal procedures	118 (84.3%)	111 (85.4%)	0.802

## DISCUSSION

According to the World Health Organization, obesity is defined as body mass index (BMI) greater than or equal to 30kg/m^2^(6). However, fat dispersion is not homogenous among patients with similar BMI. Different body types and thicker retroperitoneal fat tissue can become obstacles during percutaneous access, which is the most challenging part of the PNL procedure. Okhunov et al. developed the Size,Tract length (skin-to-stone distance), degree ofObstruction,Number of calyces involved and stoneEssence (density) (STONE) nephrolithometry scoring system for conventional PNL, which includes percutaneous tract length (PTL) and reported that STONE was predictive for stone-free status (7). In another study, Akhavein et al. demonstrated that higher residual fragments remained after conventional PNL in patients with PTL >100mm when compared with patients with PTL ≤100mm (8).

Previous studies that investigated the effect of BMI on PNL outcomes reported controversial results (9,10). When we divided patients with different BMI subgroups according to the WHO criteria, we found no significant difference in operative and post operative results. We hypothesized that SCD may have a better predictive value in forecasting outcomes of mPNL because of the variances in fat deployment. To our knowledge, this is the first study to analyze the effect of SCD on intraoperative parameters, outcomes, and complication rates of patients undergoing mPNL.

Skin-to-stone distance (SSD) is a predictive value used to estimate outcomes after SWL. Gonulalan et al. studied the significance of SSD on PNL outcomes (11). In that study, the outcomes after PNL were compared between two groups that were stratified according to their median SSDs. Gonulalan et al. did not detect a significant relationship between longer SSD and PNL success. Once adequate access to the appropriate calyx is achieved, access to the renal pelvis is relatively easier, which decreases the importance of the skin-to-stone distance. In Gonulalan’s study, nearly 45% of patients had a renal pelvis stone. Thus, we believe that SCD is more predictive than SSD in PNL procedures.

In a recent study, Cakmak et al. analyzed the effect of abdominal fat parameters on PNL outcomes (12). In a univariate analysis, they found that visceral fat area (VFA) and abdominal circumference on computerized tomography (ACCT) were predictive factors for estimating PNL success rates. Moreover, in a multivariate analysis, ACCT was found to be the only abdominal fat parameter to influence the stone-free rates. During PNL, access to the kidney is obtained through retroperitoneal fat tissue. We suggest that the skin-to-calyx distance is a more reliable parameter than the entire ACCT because patients who are obese tend to have different fat distribution patterns.

In our study, the stone-free rates for group 1 and group 2 were 82.9% and 83.1%, respectively. In the study by Knoll et al., which included patients with stones sizes similar to our study group (18 mm and 21.7mm in group 1 and 22.8mm in group 2), a stone free rate of 96% was reported following mPNL. However, Knoll et al. study only included patients with solitary renal stones (13). In another study, Kirac et al. reported a stone-free rate of 96% after mPNL (14). In that study, the rate of multiple calyx stones was 32.4%, which was comparable with our complex stone rate (42.9% in group 1 vs. 43.8% in group 2). However, the mean stone size was smaller compared with our study (10.5 vs. 21.7 mm in group 1 and 22.8mm in group 2). The stone characteristics in our study may account for our lower stone-free rates compared with other studies. Also, we found no significant correlation between the length of SCD and stone-free rates.

We found that the mean operation time and mean fluoroscopy time were significantly longer in patients with longer SCD. Ortiz et al. reported that poor fluoroscopic visualization of the stone and proper calyx in the presence of increasing retroperitoneal fat tissue may bring about difficulties in obtaining access (15). Additionally, depth perception becomes harder with increasing SCD, which results in an increased number of access trials. In our study, the number of access trials was not reported because of missing data, which will be a subject of our future studies. Keheila et al. emphasized that dilatation and securing the tract in patients with a longer SCD was a challenging and time-consuming process (16).

Fuller et al. preferred to obtain subcostal access to avoid pulmonary complications in patients who were obese and thus more vulnerable to undesired respiratory events under general anesthesia in the prone position (17). However, our approach in selecting an access location is different. Upper kidney poles are closer to the back than the lower poles and this may shorten the SCD. We performed 6 upper pole accesses in group 2, but performed no upper pole access in group 1. There was a large difference with access locations between groups but it did not reach statistical significance (p=0.051). Additionally, upper pole access was performed through the 11^th^ intercostal space in 4 patients; however, we encountered no pulmonary complications. Our results imply that using smaller caliber instruments may prevent pulmonary complications. Comparison of the efficiency and safety of upper pole access during conventional PNL and mPNL in patients who are obese may be the subject of another study.

Blood transfusion rate following conventional PNL has been reported between 0.8% and 45% (18). The incidence of blood transfusion significantly decreased with the use of miniaturized instruments. Cheng et al. reported a 1.4% blood transfusion rate in mPNL (19). Abdelhafez et al. demonstrated a 0.5% blood transfusion rate in 191 patients following mPNL (20). In our study population, blood transfusion was required in 6 (4.3%) patients in group 1, and no patients required blood transfusion in group 2. Kuntz et al. emphasized that thick perirenal fat tissue may have a protective role by providing external compression, thereby preventing hemorrhagic events intraoperatively and after removal of the nephrostomy tube, which may account for the lower transfusion rates in patients with SCD >75mm (21).

Our study, which to the best of our knowledge is the first to investigate the effect of SCD on mPNL outcomes, has some limitations. We are aware of the retrospective nature of our study. Additionally, SCD was measured preoperatively on CT scans from an axial plane, which would not match with the exact distance between the skin and the desired calyx for access. However, we measured SCD in a similar manner for every patient. Also, preoperative CT imaging was performed in the supine position and the PNL operation was performed in the prone position, which may have altered the exact SCD. Finally, our procedures were performed by different surgeons with different levels of experience.

To conclude, our study demonstrated that SCD was not a predictive factor for stone-free rates following mPNL. The SCD value >75mm was associated with a longer operation time, longer fluoroscopy screening time, and lower transfusion rates. Our findings need to be validated in further prospective, randomized studies with larger study populations.

## ABBREVIATIONS

PNL: Percutaneous nephrolithotomy
SCD: Skin to calyx distance
BMI: Body mass index
CT: Computerized tomography
KUB: Kidney-ureter-bladder X-Ray
SWL: Shockwave lithotripsy
UTI: Urinary tract infection
JJ: Double J catheter
f-URS: Flexible ureteroscopy
PTL: Percutaneous tract length
mPNL: Miniaturized percutaneous nephrolithotomy
